# Chemical genetics approaches for selective intervention in epigenetics

**DOI:** 10.1016/j.cbpa.2016.06.031

**Published:** 2016-08

**Authors:** Andrew C Runcie, Kwok-Ho Chan, Michael Zengerle, Alessio Ciulli

**Affiliations:** School of Life Sciences, University of Dundee, Division of Biological Chemistry and Drug Discovery, James Black Centre, Dow Street, Dundee DD1 5EH, UK

## Abstract

Chemical genetics is the use of biologically active small molecules (chemical probes) to investigate the functions of gene products, through the modulation of protein activity. Recent years have seen significant progress in the application of chemical genetics to study epigenetics, following the development of new chemical probes, a growing appreciation of the role of epigenetics in disease and a recognition of the need and utility of high-quality, cell-active chemical probes. In this review, we single out the bromodomain reader domains as a prime example of both the success, and challenges facing chemical genetics. The difficulty in generating single-target selectivity has long been a thorn in the side of chemical genetics, however, recent developments in advanced forms of chemical genetics promise to bypass this, and other, limitations. The ‘bump-and-hole’ approach has now been used to probe — for the first time — the BET bromodomain subfamily with single-target selectivity and may be applicable to other epigenetic domains. Meanwhile, PROTAC compounds have been shown to be significantly more efficacious than standard domain inhibitors, and have the potential to enhance target selectivity.

## Highlights

• Chemical genetics offers tools and opportunities to investigate epigenetic processes.
• Chemical probes targeting epigenetic proteins are increasingly being developed.
• Epigenetic targets pose distinct challenges to chemical genetics approaches.
• The ‘bump-and-hole’ approach allows for highly selective single-target inhibition.
• PROTACS can degrade target proteins to enhance efficacy and selectivity.

## Graphical abstract

In conventional chemical genetics, cell-active small-molecules directly block protein activity, altering phenotype. However these molecules may not be sufficiently selective or effective at modulating complex epigenetic pathways. By mutating the target protein, and creating a mutant-selective inhibitor, the bump-and-hole approach can provide single-target selectivity. PROTAC molecules direct their target to proteosomal degradation by recruiting an E3 ubiquitin ligase, resulting in more efficacious target downregulation.

**Figure:**
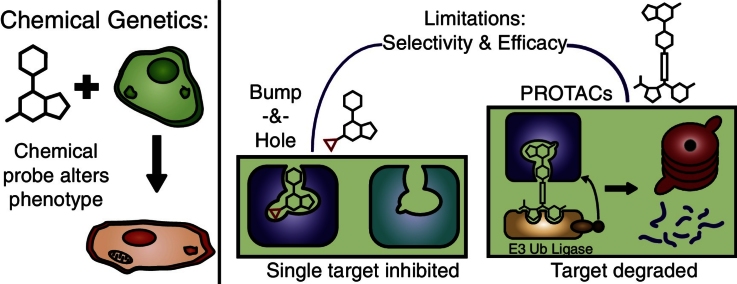

**Current Opinion in Chemical Biology** 2016, **33**:186–194

This review comes from a themed issue on **Chemical Genetics and Epigenetics**

Edited by **Danica G Fujimori** and **Stuart Conway**

For a complete overview see the Issue and the Editorial

Available online 14th July 2016

**http://dx.doi.org/10.1016/j.cbpa.2016.06.031**

1367-5931/© 2016 The Authors. Published by Elsevier Ltd. This is an open access article under the CC BY license (http://creativecommons.org/licenses/by/4.0/).

## Chemical genetics in epigenetics

Through the use of chemical probes, chemical genetics allows elucidation of the biological role and therapeutic significance of proteins [1, 2]. Chemical genetics is similar to classical genetics (knock-outs, mutations, knock-downs) [3], but alters a different point in the gene–protein–phenotype relationship. Classical genetics typically intervenes upon the gene itself (or RNA), altering or down-regulating the protein as a result; whereas chemical genetics affects the behavior of the protein directly.

Chemical genetics has several advantages over classical genetics [3]; such as reversibility, tuneability and greater spatial and temporal control. Genetic tools have additional drawbacks, such as the potential lethality of knock-outs. However, chemical probes are typically less selective than targeted gene-modification and may be active against several related proteins, preventing the connection of specific functions and phenotypes with specific proteins. Consequently, one of the greatest, and still unmet, challenges facing chemical genetics is the difficulty of generating small-molecules with exquisite single-target selectivity [2, 4••].

**Figure 1:**
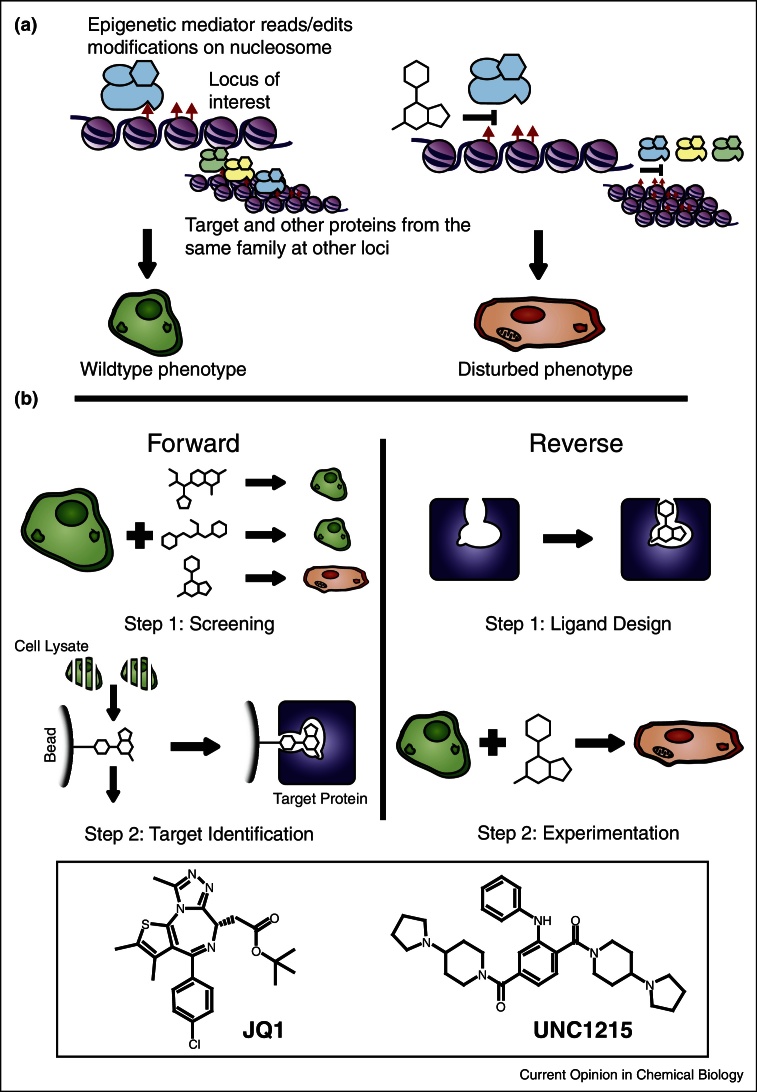
Chemical genetics, and its use in epigenetics. (a) Epigenetic protein regulates expression of multiple genes by reading, writing or erasing epigenetic marks at various gene loci. Chemical probe inhibits epigenetic protein function, altering epigenetic state of loci and the expression of relevant genes. Hence, a chemical probe facilitates the linking of the target protein to the phenotype of interest. However, linking the target and the resulting phenotype is complicated both by the target regulating multiple loci/genes and off-target inhibition of related proteins. (b) In forward chemical genetics (left panel) a library of diverse small-molecules is screened against cells. A probe is found to elicit the desired phenotype. The target protein of the probe is then identified, potentially through a chemical pulldown with a probe conjugated to beads. In reverse chemical genetics (right panel) a probe is designed and optimized for a protein of interest. This probe is then used in a variety of cells to see what phenotype it induces. Inset panel displays two examples of chemical probes targeting epigenetic proteins. JQ1 inhibits the BET bromodomains and was developed from the product of a phenotypic screen. UNC1215 targets the methyl-lysine reader L3MBTL3 and was developed through a target-driven approach.

Our growing understanding of the links between epigenetics and disease has driven the demand for well-characterised chemical tools targeting epigenetic proteins [5, 6]. Many epigenetic proteins — writers, readers and erasers of epigenetic marks [5, 6, 7] — have emerged as potential drug targets, and require chemical target validation. However, the application of chemical genetics to study epigenetic proteins faces several challenges (Figure 1a). Firstly, the difficulty in generating single-target selectivity is magnified in epigenetic systems, where many domains are clustered in large families with highly conserved substrate-binding sites [6]. This situation is similar to that of protein kinases and readers of protein phosphorylation (SH2 domains) where related proteins possess near-identical ligand-binding sites despite different functions and substrates [8, 9]. Second, probing an epigenetic target may result in complex phenotypic changes. The target may act on a large number of epigenetic marks at multiple loci throughout the genome, making it difficult to identify the gene(s) causing the phenotype of interest [10] (Figure 1a). Lastly, epigenetic regulation is highly context specific [11] and the biological effects of a chemical probe will greatly depend on the cell type and state. For a chemical genetic approach to be successful it is vital that appropriate cellular or *in vivo* models are selected to address the system of interest.

This review will illustrate recent progress and highlight novel approaches being developed to address the challenges facing chemical genetics for epigenetics.

## Forward and reverse chemical genetics in epigenetics

Both chemical and classical genetics can be described as acting in a ‘forward’ or ‘reverse’ fashion [1, 3] (Figure 1b). The forward approach involves phenotypic screens in which random mutations or diverse small-molecule libraries are employed to achieve a desired phenotype. The mutated gene, or affected protein, responsible for said phenotype is then identified. By contrast, the reverse approach begins with a known target gene or protein, the function of which is then specifically perturbed through chemical or genetic methods.

The power of forward chemical genetic approaches to study epigenetics is illustrated by many examples that have resulted in the identification of small-molecules targeting epigenetic proteins. Here we focus on epigenetic reader domains, in particular bromodomains (which recognize acetyl-lysine), but the same principles have applied to other epigenetic proteins, such as writers and erasers, including HDACs [12, 13]. Seeking a small-molecule means to suppress inflammation, a team at GSK initiated a phenotypic screen looking for up-regulators of ApoA1 [14], using a HepG2 cell line and a luciferase reporter gene. This screen identified benzodiazepine (BZD) compounds as hits, which were used in a ‘chemical pulldown’ approach to identify the bromodomains of BET (bromodomain and extra-terminal motif) proteins BRD2, BRD3 and BRD4 as targets. This led to the development of I-BET762 as a BET inhibitor [15]. In parallel, Mitsubishi Tanabe reported thienotriazolodiazepine compounds as BET bromodomain inhibitors that cause growth arrest in acute myeloid leukemia and NUT Midline Carcinoma cell lines [16]. Further work by Bradner, the SGC and collaborators pursuing a potential NUT-midline carcinoma treatment, led to the report of JQ1 (Figure 1b) as a pan-selective BET inhibitor [17]. These discoveries demonstrated the potential of phenotypic screens to identify important roles of epigenetic mediators. They also highlighted the druggability of BET proteins and started a new wave of interest in the science community using these chemical tools to probe for BET functions.

The important biological roles of epigenetic proteins, coupled with the success of phenotypic screens at linking these targets to disease, have motivated growing efforts to identify their endogenous substrates and de-orphanize them of small-molecule probes. Following the discovery of JQ1 [17] and I-BET762 [15], several inhibitors targeting BET and other bromodomains have been developed, guided by an abundance of high-resolution crystal structures. Said inhibitor design has recently been comprehensively reviewed [18, 19, 20]. Reverse chemical genetics has also been employed to target readers of methyl-lysine marks such as chromodomains, PHD fingers and MBT domains [21, 22, 23, 24]. While most of these probes have not yet been used to address biological questions, in some cases they have revealed interesting biochemical insights. For example, UNC1215 (Figure 1b, a selective probe for the methyl-lysine reader L3MBTL3) has suggested a possible polyvalent method of substrate recognition [25] and revealed that L3MBTL3 functions as a dimer [26].

## Chemical probes as chemical tools

Chemical probes have been used to investigate the function and importance of the BET proteins in a wide range of contexts, from cancer and inflammation to neurology and reproductive biology. These studies have highlighted the role that BET proteins play in processes, such as cell growth and differentiation [27^•^], through the modulation of many signaling pathways such as C-MYC, NF-kB and the Jak/STAT pathway. The wide range of cell-types and tissues these compounds have proven useful in is itself evidence for the benefits of making chemical probes available to as wide a range of researchers as possible. These compounds are pan-selective BET inhibitors, which prevents specific phenotypes being linked to a specific BET protein or bromodomain. This has been compensated for somewhat by the use of complimentary genetic tools (KO, RNAi) to identify the relevant protein [28, 29, 30]; and by a mutant-sensitive chemical genetic approach [31^••^], discussed later. Some selectivity within the BET subfamily has been reported [32, 33, 34, 35] but said probes still lack single-target specificity.

**Figure 2:**
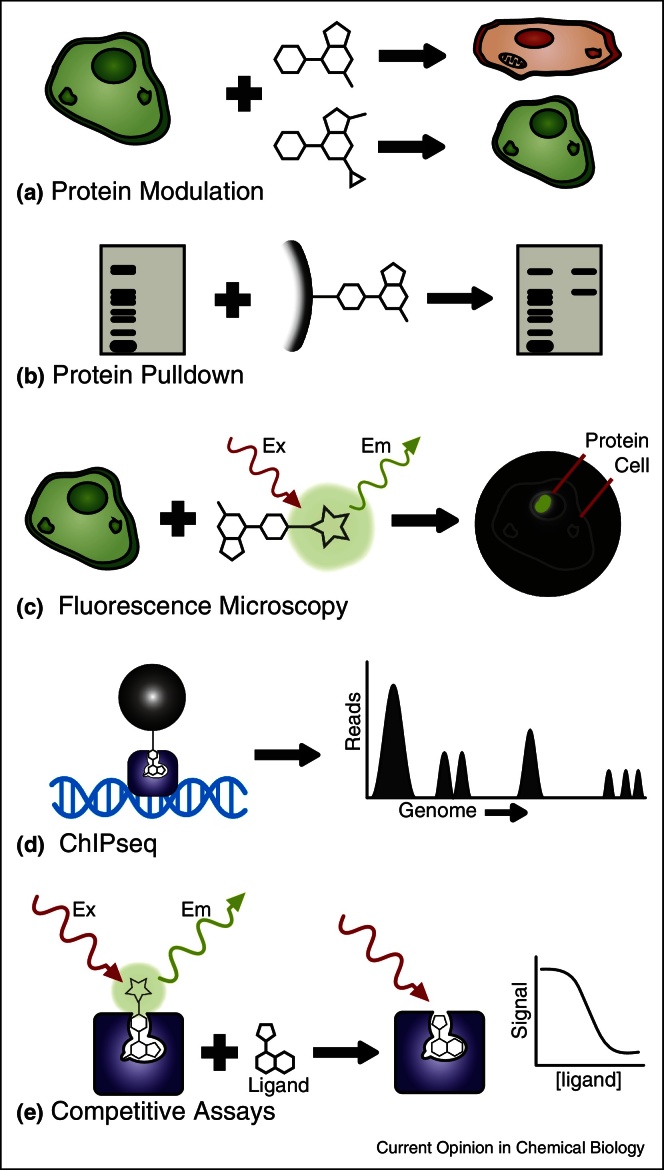
Chemical probes as chemical tools. (a) Chemical probes used *in vivo* to investigate protein function. Inactive analogs can be used to control for off-target effects. (b) Chemical probe conjugated to bead, for use in protein pulldown (shown via western blot). (c) Fluorescent probe used for fluorescence microscopy, showing cellular localization of target. (d) Probe with affinity tag (e.g. biotin-streptavidin) used in ChIPseq experiment, showing probe localization along genome. (e) Modified probe used in competitive binding assay (e.g. fluorescence polarization), for library screening.

Chemical probes can be used for many applications beyond mere target inhibition (Figure 2). Compounds bearing an affinity tag can be used for chemical pulldowns of the target protein [14, 36•]. These pull-downs can also identify splice-variants of the target protein, the subunits it may associate with and potentially related proteins with sufficiently similar binding sites. These ‘pulled down’ proteins can then also be analyzed for the presence of post-translational modifications. A chemical pulldown using the probe UNC1215 revealed an interaction between L3MBTL3 and BCLAF1 [25], while UNC0965 (a biotinylated G9a probe) gave a higher pulldown signal/noise ratio than a G9a antibody [37]. Recently, a UV-triggered JQ1 cross-linker was developed [38], and used to identify potential off-targets of JQ1.

Anders *et al.* [36^•^] designed a biotinylated JQ1 probe for use in a ChIPseq experiment, and were able to show the localization of JQ1 and its targets throughout the chromosome. The JQ1 cross-linker mentioned previously [38] has also been used to conjugate BET proteins to a fluorophore, for use in live-cell fluorescence microscopy. Fluorescent probes provide an alternative to the established practice of using GFP-fusion constructs in microscopy and may prove advantageous, such as when the target protein is not genetically pliable or the GFP tag impacts the wild-type protein function.

## Advanced chemical genetics

The use of chemical probes, both in forward and reverse chemical genetics, has revealed much about the function of epigenetic proteins, but has traditionally been restricted to a conventional target:inhibitor modality. More advanced forms of chemical genetics are being developed that offer new opportunities to bypass inherent limitations of target inhibition and provide an additional level of biological insights.

### Bump-and-hole approach

**Figure 3:**
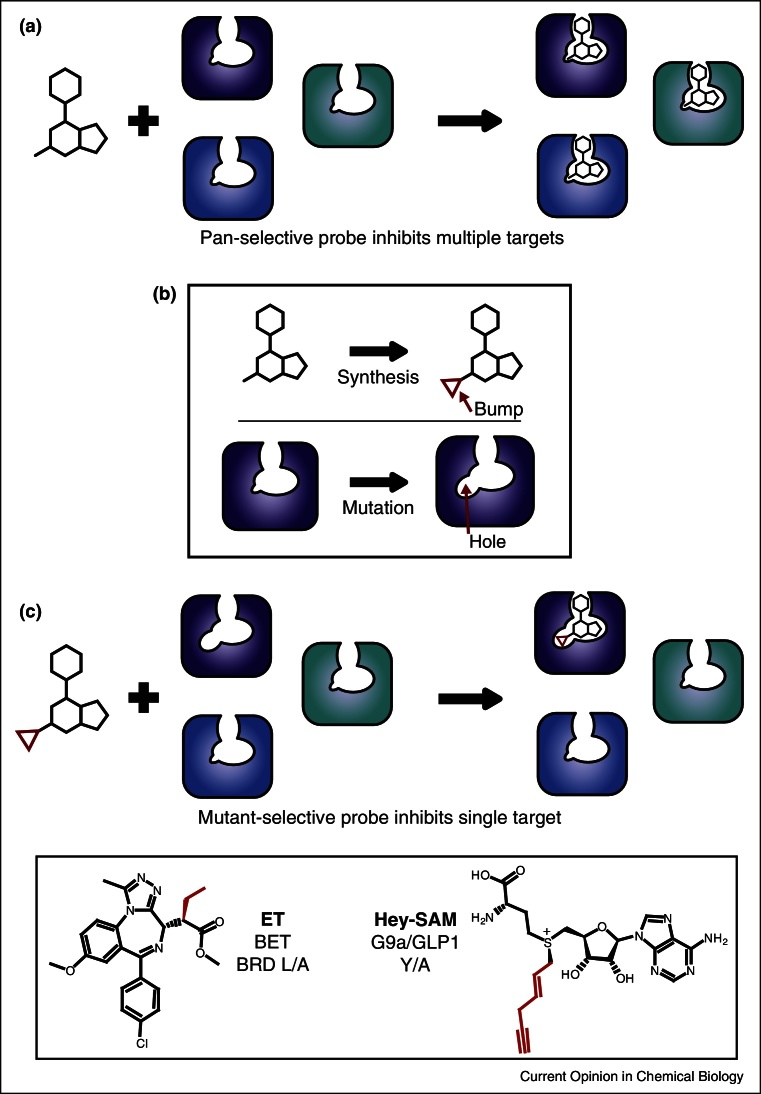
Selective target inhibition via bump-and-hole. (a) Pan-selective probe binds multiple related proteins. This generates a (potentially complex) phenotypic change. (b) Chemical probe is modified to incorporate a ‘bump’, and target protein is mutated to introduce a ‘hole’. (c) Bumped probe binds only the mutated target, allowing the resulting phenotypic change to be connected to a single protein. Inset panel illustrates two successful examples of bumped molecules developed against mutated epigenetic domains.

To overcome the challenge of achieving selectivity against homologous, highly similar binding sites, an orthogonal protein:ligand pair can be generated. In the so-called bump-and-hole approach [39^•^], which has found attention early on with cofactor-dependent enzymes [40, 41], a small hole within the binding site is generated by site-directed mutagenesis. An allele-specific probe can then be obtained by introducing a compensatory bulky modification to the ligand. This chemical group will be expected to produce steric clashes and abolish binding to the wild-type protein, but be accommodated by the mutant (Figure 3).

The bump-and-hole technique has been applied to epigenetic enzymes and their cofactor ligands as a mean to identify their specific substrate proteins. For example, the methyl-donor SAM (*S*-adenosyl-*l*-methionine), can be modified at various positions by introducing a sterically demanding group which must then be accommodated by a PMT (protein methyl transferase) enzyme. Modification on the adenosyl-N^6^ position allowed study of the methylation of substrates by yeast RMT1 [41], and by modifying the 2′-hydroxyl and 3′-hydroxyl groups of the ribose unit of SAM the enzymatic activity of the histone methylase vSET was studied [42]. In another approach PMTs were engineered to process methyl sulfonium SAM-analogs allowing the enzyme to transfer distinct chemical groups (e.g. alkyne units) to the substrate that could then be used to pulldown target proteins [43, 44, 45]. This helped to identify the genome-wide chromatin-modifying activities of G9a and GLP1, through next generation sequencing of the enriched pulldown samples of chromatin DNA [45]. A similar approach was applied to KATs (lysine acetyltransferases) where synthetic Acetyl-CoA surrogates where used to label KAT cellular targets [46].

This technique can also be used to create chemical probes with single-target selectivity. Baud *et al.* [31^••^] succeeded in creating a derivative of the BET inhibitor I-BET762/JQ1 selective for BET bromodomains possessing a distinct leucine/alanine mutation. Importantly, this selectivity was controllable and achieved over the entire BET subfamily. Using this approach it was shown that small-molecule targeting of the N-terminal bromodomain alone is sufficient to displace BRD4 from chromatin [31^••^]. This marks both the first time that single BET proteins and their individual bromodomains can be targeted selectively by a small-molecule and also the first example of the bump-and-hole approach being applied to a protein–protein interaction. This approach may be applicable to other bromodomains or epigenetic reader domains, allowing links between specific proteins and phenotypes to be shown with greater confidence than before.

Application of the bump-and-hole approach faces several challenges. Both the nature of the mutation and the nature of the bump must be balanced to find a compromise between two extremes. More sizable mutations to generate larger ‘holes’ will probably make selective ligands easier to generate. However these mutations may disrupt the structure and function of the target protein, complicating the interpretation of results and potentially rendering mutant cell lines non-viable. The ‘bump’ meanwhile must be balanced between potency, selectivity and physiochemical properties. Larger and more sterically demanding modifications will potentially have a greater impact on reducing wild-type inhibition but could also reduce the potency against the mutant proteins. Bulkier bumps will also make the compound more lipophilic, a trait associated with pharmacokinetic liabilities. It is not currently understood how broadly applicable the bump-and-hole system may be. Results with BET bromodomains show that not all binding site residues can be mutated to achieve acceptable outcomes [34], with a conserved leucine residue from the ZA loop [31^••^] being the first one to both allow single-target selectivity and avoid fatally disrupting the protein's endogenous function.

### PROTAC approach

**Figure 4:**
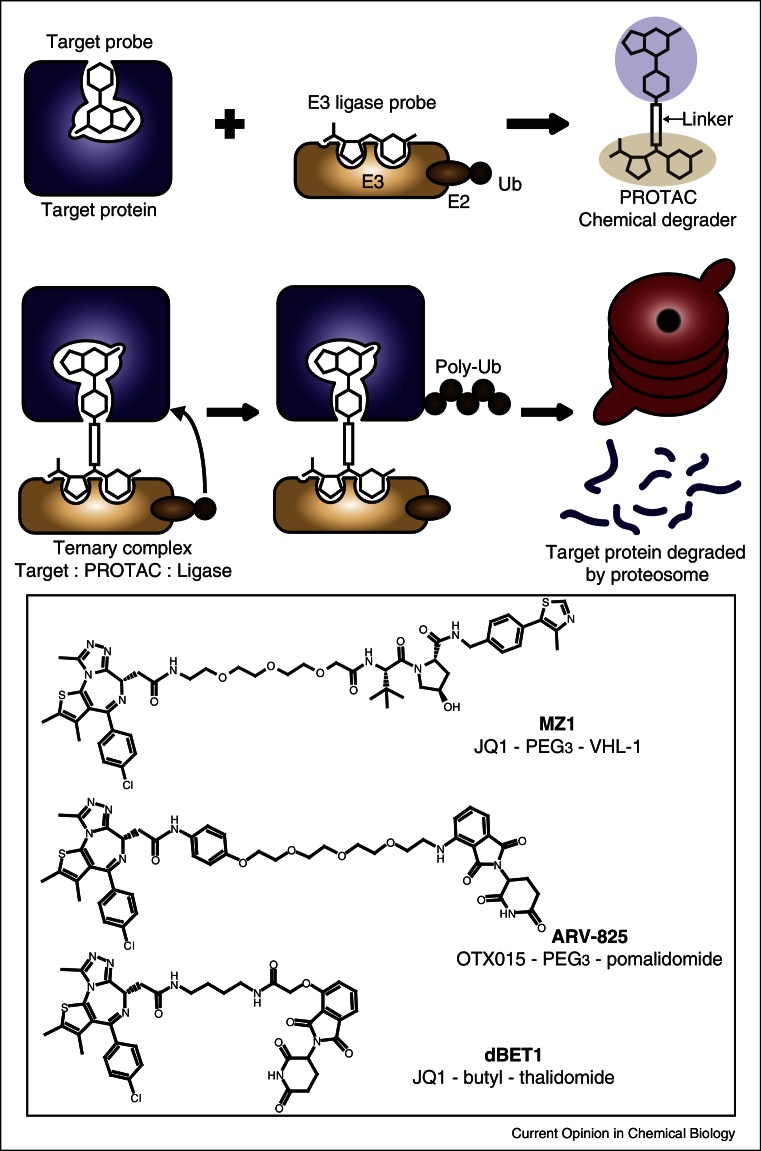
Chemically induced protein degradation via PROTAC. A chemical probe targeting the protein-of-interest and a probe targeting an E3 Ubiquitin ligase are connected by a linker, forming a PROTAC molecule. This PROTAC then binds both the target and ligase proteins, forming a ternary complex in which the target is ubiqutinylated. The polyubiquitinylated target is then degraded by the proteasome. The inset panel illustrates successful examples of PROTAC molecules targeting BET proteins for degradation by recruiting E3 ligases such as VHL (MZ1) and cereblon (ARV-825 and dBET1).

Small molecules can be designed to induce intracellular protein degradation, leading to the destruction of a target protein. These so-called PROTACs (proteolysis targeting chimeras) [47] are heterobifunctional compounds consisting of a moiety binding an E3 ubiquitin ligase linked to another that binds the target of interest, thus marking the target for degradation by the proteasome (Figure 4). Complementary approaches for targeting proteins for degradation have employed introduction of hydrophobic degrons such as adamantyl moieties or Boc-protected arginines [47].

Unlike conventional inhibitors, PROTAC molecules act substoichiometrically via a catalytic mechanism [48]. As a result, the concentration required for PROTACs to be active in cells tend to be lower compared to those needed to be reached and maintained with inhibitors, leading to fewer off-target effects and a more selective chemical intervention on the desired target. Another attractive feature of this advanced form of chemical genetics is that it achieves a chemical knock-down directly at the posttranslational level. This intervention is expected to phenocopy more closely the effect of conventional genetic knock-out and knock-down strategies, but without interfering directly on the DNA or RNA. Finally, PROTACs can function regardless of where on the target protein they bind. Unlike conventional inhibitors they do not need to bind to a functional site on the target protein, which may aid in the targeting of poorly druggable protein–protein interactions that are common in epigenetic systems.

The PROTAC approach has recently been applied successfully to the field of epigenetics, with three complementary studies demonstrating effective targeting of BET proteins [49••, 50••, 51••]. Using JQ1 and high-affinity ligands that had been designed against the VHL (von Hippel-Lindau) E3 ligase, our laboratory developed a series of PROTACs that exhibited rapid, reversible and long-lasting destruction of BET proteins [49^••^]. Interestingly these compounds could induce potent and preferential removal of Brd4 over a suitable concentration window, leaving the homologous Brd2 and Brd3 relatively untouched and leading to more Brd4-specific response in cancer cells [49^••^]. The most potent compound, MZ1, was active at a sufficiently low concentration not to induce stabilization and transcriptional activation of HIF-1α, the natural substrate of VHL. In parallel studies, Winter *et al.* [50^••^] and Lu *et al.* [51^••^] tethered the same BET inhibitor scaffold to a ligand for a different E3 ligase, cereblon, yielding highly potent pan-BET degraders dBET1 and ARV-825, respectively. BET-degrading PROTACs exhibited superior antiproliferative efficacy in cellular and mouse models of c-MYC driven lymphomas and leukemias compared to their parent BET inhibitors [50••, 51••].

Together, these important studies demonstrate that induced target degradation can elicit a more pronounced and potentially more target-specific biological response than domain-targeted chemical inhibition. In particular, the reported VHL-based BET PROTACs provide first proof-of-concept for turning non-selective or pan-selective inhibitors into more selective degraders.

The design and practical implementation of PROTACs pose unique challenges. Because of their higher molecular weight PROTACs would be expected to display poorer DMPK properties than their smaller constituent ligands (for example faster metabolic clearance). These potential liabilities could impair efficacy *in vivo* and limit their therapeutic development. Assuming that PROTAC linker domains are not involved in ligase or target binding, different forms of chemical linkers could be used to fine-tune pharmacokinetic properties in addition to target degradation efficacy. Another difficulty in PROTAC design is the lack of a practical, rapid and robust assay for measuring target-degradation activity beyond low-throughput western blotting or expensive chemoproteomics by mass spectrometry. Furthermore, *in vitro* ubiquitylation assays are typically low-throughput and non-quantitative. On the other hand, the affinity of PROTAC compounds for purified target or ligase proteins can be readily obtained using conventional biophysical/biochemical techniques, and ternary-complex formation can potentially be quantified through AlphaScreen [50^••^] or FRET techniques [52]. Finally, PROTACs suffer from the so-called ‘hook-effect’ [49••, 51••] wherein at high concentrations formation of binary complexes (PROTAC:target and PROTAC:ligase) competes with and eventually surpass the formation of the productive ternary complex.

## Conclusion and future perspectives

Chemical genetics can help to elucidate and understand the function of epigenetic proteins and their role and significance in disease, but epigenetic targets pose distinct challenges. Many attractive epigenetic proteins remain to be successfully probed by a small molecule, suggesting their inherently low druggability. Conversely, within those families that have proven druggable, such as HDACs and bromodomains, issues of target selectivity remain to be addressed. In future it will be important to improve upon and extend beyond the established small-molecule targeting paradigm, that is, single domain-focused chemical inhibition. More advanced and sophisticated ways of carrying out chemical genetics, such as the bump-and-hole and PROTAC approaches, have shown to allow for more selective targeting and improve on chemical probe efficacy. We anticipate that combining these advances in chemical genetics with the recent developments in genetic tools such as CRISPR-Cas9 gene editing and refined ChIP-Seq methods for genome-wide mapping will truly enable scientists to push the frontiers of the field, ultimately increasing the level and confidence of epigenetic target validation.

## References and recommended reading

Papers of particular interest, published within the period of review, have been highlighted as:

• of special interest
•• of outstanding interest
